# Site Fidelity and Individual Variation in Winter Location in Partially Migratory European Shags

**DOI:** 10.1371/journal.pone.0098562

**Published:** 2014-06-03

**Authors:** Hannah Grist, Francis Daunt, Sarah Wanless, Emily J. Nelson, Mike P. Harris, Mark Newell, Sarah Burthe, Jane M. Reid

**Affiliations:** 1 Institute of Biological and Environmental Sciences, University of Aberdeen, Aberdeen, Aberdeenshire, United Kingdom; 2 Centre for Ecology and Hydrology, Penicuik, Midlothian, United Kingdom; 3 Scottish Ornithologists' Club, Aberlady, East Lothian, United Kingdom; Institute of Ecology, Germany

## Abstract

In partially migratory populations, individuals from a single breeding area experience a range of environments during the non-breeding season. If individuals show high within- and among- year fidelity to specific locations, any annual environmental effect on individual life histories could be reinforced, causing substantial demographic heterogeneity. Quantifying within- and among- individual variation and repeatability in non-breeding season location is therefore key to predicting broad-scale environmental impacts on the dynamics of partially migratory populations. We used field resightings of colour-ringed adult European shags known to have bred on the Isle of May, Scotland, to quantify individual variation and repeatability in winter location within and among three consecutive winters. In total, 3797 resightings of 882 individuals were recorded over 622 km of coastline, including the Isle of May. These individuals comprised over 50% of the known breeding population, and encompassed representative distributions of ages and sexes. The distances from the Isle of May at which individuals were resighted during winter varied substantially, up to 486 km and 136 km north and south respectively and including the breeding colony on the Isle of May. However, resighting distances were highly repeatable within individuals; within- and among-winter repeatabilities were >0.72 and >0.59 respectively across the full September-March observation period, and >0.95 and >0.79 respectively across more restricted mid-winter periods. Repeatability did not differ significantly between males and females or among different age classes, either within or among winters. These data demonstrate that the focal shag population is partially migratory, and moreover that individuals show highly repeatable variation in winter location and hence migration strategy across consecutive winters. Such high among-individual variation and within-individual repeatability, both within and among winters, could lead to substantial life history variation, and therefore influence population dynamics and future conservation management strategies.

## Introduction

It is increasingly recognised that demographic heterogeneity, stemming from temporal and spatial variation in individual life-histories, can substantially influence population dynamics [1]–[3]. Such life-history variation can in turn be caused by combinations of intrinsic factors including genetic and maternal effects, and by extrinsic environmental effects [4], [5]. In particular, spatial heterogeneity in environmental conditions can cause considerable life-history and demographic variation, both within and among populations. For example, the growth of stochastically-dispersing seeds depends on the physical characteristics of colonised sites [6] and small-scale environmental variation affects individual reproductive success in vertebrates [7]–[9]. Such environmental effects can also extend beyond immediate life-history components and have carry-over [10] or delayed effects [11] on future reproductive success and survival, with further consequences for population dynamics [5], [12], [13]. One critical step towards understanding patterns of life-history and demographic heterogeneity and their population dynamic consequences is therefore to quantify variation in the locations occupied by population members, and by extension the environments experienced.

Many previous studies have considered the life-history consequences of spatial variation in the environments that individuals experience during the breeding season [7], [10]. However, for iteroparous species, the non-breeding season can encompass over half the annual cycle and is often the period of highest mortality, which can strongly influence population growth rate [14], [15]. In migratory populations, individuals experience entirely different environments during the breeding and non-breeding seasons. Furthermore, individuals from a single breeding population can occupy a diverse range of non-breeding locations or habitats [16], [17], potentially creating within-population life-history variation through environmental effects [18], [19]. In the case of partially migratory populations, where some individuals migrate while other individuals remain resident at the breeding location, among-individual variation in environmental conditions experienced can be particularly marked [20].

If individuals show strong within- or among- year fidelity to specific non-breeding locations and/or consistency in migratory strategy, any annual environmental effect can be reinforced [17], [21]. The combination of among-individual variation and within-individual repeatability in non-breeding location could therefore cause substantial variation in individual fitness [17], [22], create population structure due to non-random segregation of individuals across locations and environments [19], [23], and shape population dynamics through site-dependent regulation [24], [25]. The pattern of individual fidelity to specific non-breeding locations might also guide the most effective approach to site-based conservation management, through highlighting areas that underpin population persistence. However, the ultimate population dynamic consequences of individual variation in non-breeding location and site fidelity also depend on whether resulting demographic variation is structured or random, particularly if fidelity varies with intrinsic factors such as age, sex or condition [12], [26]. Quantifying within- and among- individual variation in location both within and among seasons, and any underlying structure in this variation due to intrinsic factors, is therefore key to understanding composite environmental effects on population dynamics and applying this knowledge to effective conservation policy.

However, despite the value of quantifying individual variation and repeatability in location across seasons, relatively few studies have yet tracked individuals to precise locations across both breeding and non-breeding seasons across multiple years in sufficient numbers to allow robust population-wide inferences of potential demographic heterogeneity. Some studies have been able to assign known breeding individuals to winter locations based on single captures or using indirect methods such as stable isotope ratios, but these methods assume that individuals remain in a single location throughout the winter [22], [27]. New tracking technologies such as satellite tags and geolocators have revolutionised our ability to identify winter locations and associated environmental effects on individuals, particularly seabird species which are often used as marine ecosystem bioindicators but can be difficult to study outside the breeding season [28]. These approaches have demonstrated that separate breeding populations mix during winter [29], [30], that populations use much larger areas than previously thought [31], and that individuals are flexible in winter location between years [32]. However, while such technologies can be invaluable for quantifying fine detail of individual movements, sample sizes of individuals are thus far relatively small, data are often only retrieved for individuals that survive and return to breed and can therefore be recaptured, or are restricted to single years [33]. One complementary approach to locating individuals within and among seasons is therefore through resightings of permanent field-readable markings which, in appropriate systems, can provide location data for large numbers of individuals across multiple years [17].

We used large-scale field resightings of colour-ringed European shags (*Phalacrocorax aristotelis*; hereafter “shag”) breeding at a known colony to quantify within- and among- individual variation in winter location. We aimed to determine if individuals could be resighted across a range of locations including the breeding colony during the winter period, which would indicate the population is partially migratory. We tested whether individuals were repeatable in winter resighting location within and among years, and therefore show site fidelity over the winter over a range of geographical locations. Finally, we investigated whether patterns of site fidelity varied across different ages and sexes.

## Methods

### Ethics Statement

The Isle of May is a Scottish Natural Heritage (SNH) National Nature Reserve (NNR), a Special Protection Area (SPA) and a Site of Special Scientific Interest (SSSI). The island is owned by SNH, and all work was approved under research licences issued by SNH. Capture of shags at nest sites and fitting of metal and colour rings were carried out under licence from the British Trust for Ornithology (BTO). Ringing took <2 min, after which adults were released close to the breeding site and chicks were returned to nests. Adult shags invariably returned to normal breeding activities within a few minutes. Winter resightings were made from publicly accessible locations and from sufficient distance to ensure that shags were not disturbed.

### Study system

**Figure 1:**
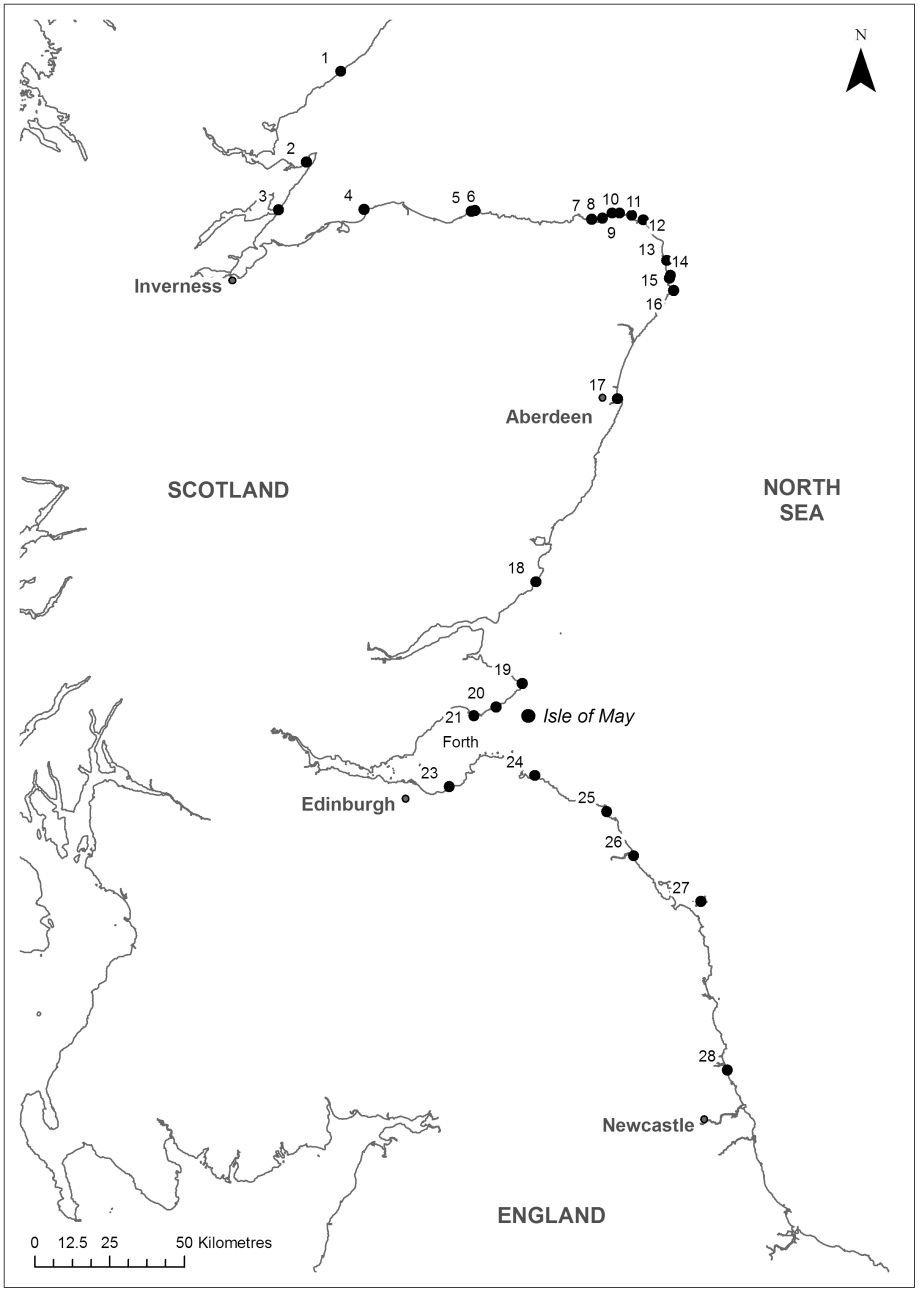
Winter survey sites for colour-ringed shags. Black points denote positive survey sites, where at least one adult colour-ringed shag known to have bred on the Isle of May was resighted in at least one winter 2009–2012. Sites are defined as roosts separated by ≥1 km.

European shags are large, pursuit-diving seabirds distributed principally across north-west and southern Europe [34]. In 1999–2002 ca. 29,000 pairs bred across the rocky coastlines of the UK, with the largest colonies found in the north and west, including 734 pairs on the Isle of May National Nature Reserve, an island in the outer Firth of Forth, Scotland [34]. Since then, numbers have declined, both in the UK and on the Isle of May, where the population varied between 465–505 occupied nests in 2009–2011 [35]. The demography and ecology of the Isle of May (56°11′N 2°33′W, Figure 1) breeding population has been studied since 1961 [36]–[39]. Shags breeding on the Isle of May are listed under European Special Protection Area (SPA) legislation [40] and are of amber conservation concern in the UK [41]. However, relatively less is known about the movements of the population during the non-breeding (winter) season, or the potential impact of variation in winter environmental conditions on individual life-histories and therefore population dynamics [42].

Each year since 1997, shag nest sites on the Isle of May were systematically monitored and chicks surviving to ca. 20 days post-hatch were ringed with British Trust for Ornithology (BTO) metal rings and coloured plastic rings engraved with unique three-letter codes, which are readable in the field at distances up to 150 m using optical equipment. In addition, unringed adults (i.e. immigrants or individuals not ringed as chicks) were colour-ringed, breeding adults identified, and any worn or damaged rings replaced [43]. Since 1997, 692 adults and 11530 chicks have been colour-ringed.

On the Isle of May, shags can first breed aged two years [37], although normally not until a year or more older, and typically survive for multiple breeding years (mean annual adult survival probability  = 0.88±0.02SE) [38], [42]. They typically show high natal philopatry: 90% of shags hatched on the Isle of May that were found breeding during large-scale surveys of eastern North Sea colonies during 2008–2010 were breeding on the Isle of May [42]. Shags also show high breeding philopatry to their colony, and to breeding sites within the colony [39], [42], [44]. Annual breeding is normal, but in some years a proportion of adults fail to breed [45].

European shags are thought to be largely sedentary across much of the species' range, although some populations and/or individuals are migratory [36], [46]. Broad-scale geolocator data suggest that individuals breeding on the Isle of May remain around northern North Sea coasts year-round [47]. Furthermore, BTO-ringed shags from the east coast of Scotland that were subsequently recovered dead were found a median recovery distance of <100 km from the colony [36], [48].

### Winter resightings

In order to quantify individual variation and repeatability in winter location, colour-ringed shags were resighted across a geographical range that is likely to encompass most of the Isle of May breeding population's winter range. Historical dead recoveries of shags ringed in the Firth of Forth, including the Isle of May, were used to define the total geographical range likely to be relevant to Isle of May shags in winter as the east coast of Scotland and northern England [36], [42], [48]. However, as these recoveries included individuals breeding at unknown colonies, they describe the broad geographical distribution of winter mortality across a meta-population rather than specific winter locations of live adults breeding at any single colony.

Shags have a partially wettable plumage that requires individuals to return to shore regularly to dry out and thermoregulate [49]. This restricts their distribution to coastal areas and, unlike truly pelagic seabirds, means that ringed individuals can be resighted on land year-round. Pilot fieldwork undertaken during the winter of 2008–2009 confirmed that shags colour-ringed on the Isle of May could be located across a large geographical range in winter using field resightings: 307 colour-ringed individuals were observed across 55 surveys spanning 540 km north to 355 km south of the Isle of May, covering the full geographical range highlighted by previous dead recoveries and suggesting that at least some individuals migrate over a wide range. Both night and day roosts were identified, where shags congregate overnight and during daytime foraging breaks respectively, including on cliffs, offshore skerries, and harbour walls. Day roost occupancy varied markedly with time of day, tide and weather, and decreased in mid-winter as the proportion of daylight hours spent foraging increased [47].

For survey purposes, each known shag day or night roost separated by >1 km was defined as a separate “site”. Following the pilot fieldwork, winter surveys were undertaken across known accessible sites during 1^st^ September to 31^st^ March 2009–2010, 2010–2011 and 2011–2012. As well as sites visited during the pilot fieldwork, additional sites were identified by consulting local birdwatchers and bird reports, and by further field exploration. The primary aim was to repeatedly locate individuals wintering across a large geographical range, not to map or infer the winter distribution of the entire population. Consequently, surveys were structured to ensure that multiple key sites were visited repeatedly within and across winters, approximately every 1–2 weeks, with visits strategically planned to coincide with site-specific times, tides or weather conditions to maximise resighting probabilities. In addition, ad hoc resightings at regularly surveyed sites and at any unsurveyed sites were actively solicited from birdwatchers in order to ensure individuals could be resighted across a diverse range of locations. In total, 21 sites were regularly surveyed, and *ad hoc* sightings were recorded at seven further sites scattered across the full geographic range, covering 486 km north and 136 km south of the Isle of May and including the Isle of May itself (Figure 1). South of this range shags are uncommon since the coast has few potential roost sites.

During each survey, at least one observer observed the roost with a 60× magnification telescope for 30 mins to 5 hours depending on the number and turnover of shags, continuing until all visible individuals were checked for rings, or light, tide or weather conditions prevented further resightings. Individual ring codes were recorded and rechecked by a second visual sweep. Not all areas at each site where shags roosted were accessible and not all colour-rings were fully visible. The probability of resighting a ringed individual that was present was therefore <1, and presumably varied among sites and surveys. Survey effort and efficiency varied across the three winters due to variation in weather and increasing knowledge of roost sites and patterns of tidal and diurnal use, but this variation does not impede the current objectives of quantifying individual variation and repeatability in winter location across a sample of individuals (see Discussion). The spatial and temporal distribution of resightings is further described in the supporting information (Figures S1–S5).

### Data structure

The final dataset comprised all resightings of adult colour-ringed shags known to have bred on the Isle of May. Locations where shags were resighted away from the Firth of Forth (Figure 1) were coded as a linear coastline distance (km) away from the Isle of May using an equidistant projection in ArcGIS v. 10.1, with southerly and northerly distances coded as negative and positive respectively [50]. Since shags typically do not cross land or long stretches of open sea, coastline distance is more biologically relevant than Euclidean distance for most locations [42]. However, locations within the Firth of Forth were directly across short stretches of water (Figure 1) and therefore coded as Euclidean distance from the Isle of May.

To quantify overall resighting success per winter, the proportions of colour-ringed adult shags that were observed breeding on the Isle of May during a summer that were resighted the following winter were calculated. To verify that the shags resighted during each winter were broadly representative of the Isle of May breeding population, the sex and age distributions of the resighted shags were compared to those of colour-ringed individuals breeding on the Isle of May the previous summer using chi-squared tests. Breeding shags were sexed at the nest based on voice and behaviour [51], meaning that most adults resighted in winter were of known sex, and individuals that had been ringed as chicks were of known age. The numbers of individuals aged ≥15 years were too small to be compared directly and so were pooled, and individuals first ringed as adults were assigned an age of three years (the modal age of first breeding) in their first recorded breeding season for comparative purposes [44].

### Defining winter

Quantifying individual variation in winter location requires the period of ‘winter’ to be defined. This is not straightforward in populations that undertake partial or short-range migrations, because there is no clear date by which all individuals have left the breeding grounds, and there can be extended migration periods with substantial individual variation in departure and return dates [52]. Resightings spanned 1^st^ September to 31^st^ March each winter, and consequently included pre- and post-migratory periods for different individuals across both autumn and spring migrations. An objective definition of ‘winter’ was therefore required to ensure that individual repeatability in winter location could be evaluated without downward bias due to including resightings of individuals before or after their migration, or upward bias due to eliminating resightings of individuals that were genuinely mobile within or among winters. Resightings of individuals that migrated from the Isle of May to a different winter site during the resighting period were therefore used to define a series of nested ‘winter’ periods at the population level.

**Figure 2:**
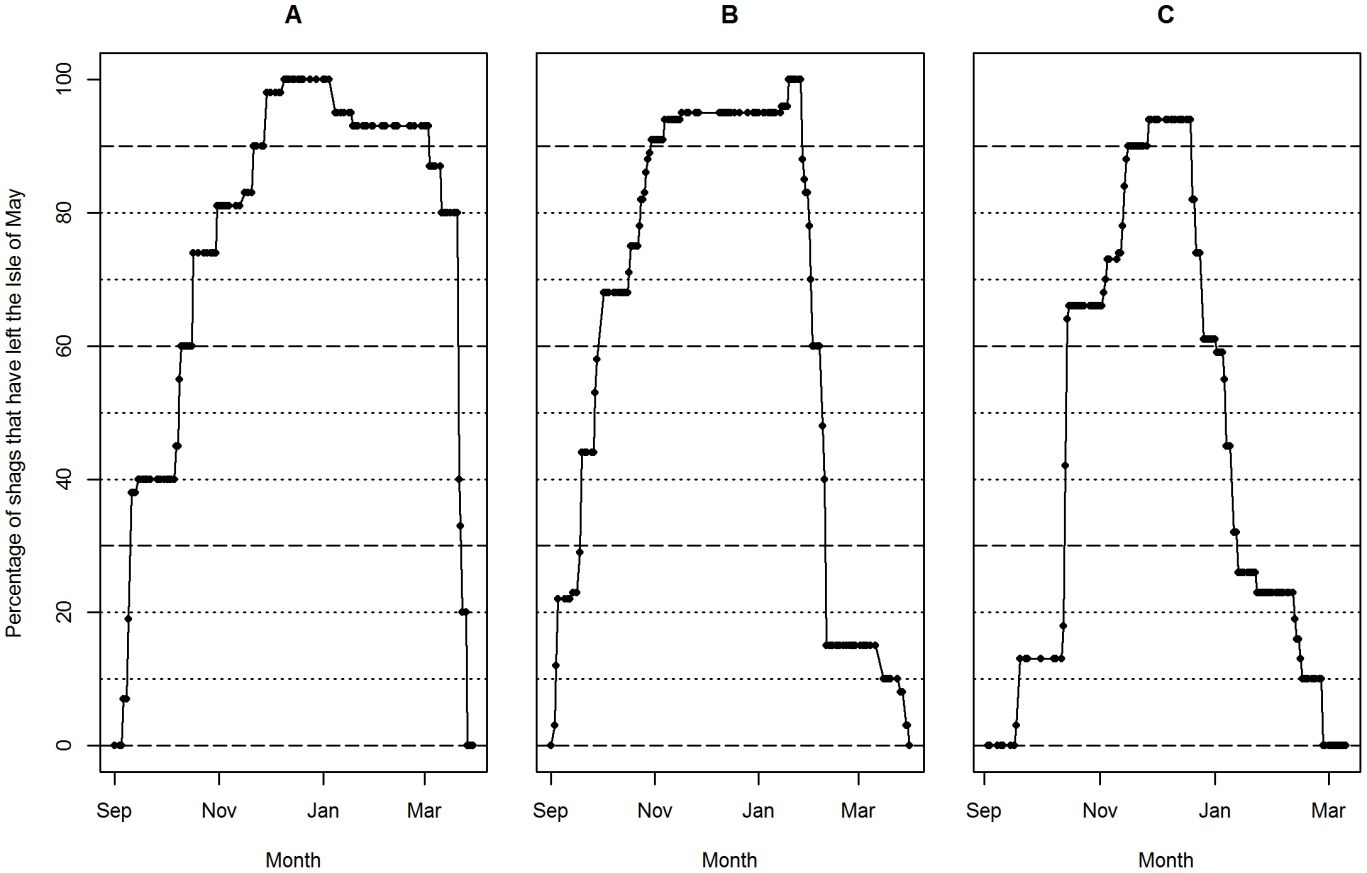
Movement boundaries. The percentages of colour-ringed adult shags known to have bred on the Isle of May that were resighted on or near the Isle of May and at an alternative winter site during winters A. 2009–2010, B. 2010–2011, and C. 2011–2012 that were away from the Isle of May on progressive dates. Dotted lines indicate 10% intervals, dashed lines denote the movement boundaries used to subset the data for repeatability analyses (0%, 30%, 60%, 90%).

Individual shags were considered to still be at or have returned to the breeding colony if they were resighted on the Isle of May or at an associated day roost in the Firth of Forth (Figure 1). Across all three full winter field seasons, 237 individuals were initially resighted on or near the Isle of May and subsequently resighted at a different site, and 89 individuals were resighted at a different winter site and then back on or near the Isle of May towards the end of the same winter. These individual movements were used to estimate time intervals (hereafter “movement boundaries”) when specific percentages (from 0% to 90% in 10% increments) of individuals had departed from or returned to the Isle of May, taking the last autumn and first spring dates on which an individual was observed on or near the Isle of May as the departure and return dates respectively (Figure 2). The resulting movement boundaries are properties of the current datasets, given patchy observation dates at current survey sites, and may not reflect the migration dates of the entire population.

### Variation and repeatability

To quantify the degree to which individual shags were resighted at consistent winter locations both within and among winters, individual repeatabilities (R) in winter location were estimated by fitting linear mixed effects models with random individual effects and no fixed effects. The dependent variable was the distance from the Isle of May in kilometres of each individual resighting, and therefore measured continuous variation in location. The estimated variance components were used to calculate the intra-class correlation coefficient as the ratio of among-individual variance to the total variance [53], [54].

To quantify within-winter repeatability (R_w_), the dataset was restricted to individuals resighted on ≥2 dates within each movement boundary within each winter [53], and each of the three winters was analysed separately. The probability of obtaining the observed R_w_ by chance (i.e. if individuals moved randomly across the observed geographical range) was estimated by shuffling all locations across resightings of all individuals without replacement, recalculating the repeatabilities within each of 10,000 iterations and comparing observed R_w_ to the simulated null distribution.

To quantify among-winter repeatability (R_a_), the dataset was restricted to individuals that had been resighted in ≥2 winters. Within each winter a single resighting was randomly selected for individuals that were resighted on ≥2 dates, and mean R_a_ and 95% sampling intervals were calculated across 1000 iterations. The probability of obtaining the observed R_a_ by chance was additionally estimated by shuffling the entire dataset as for R_w_ above. For both R_w_ and R_a_, repeatabilities were recalculated across nested subsets of the data defined by the movement boundaries (0, 30%, 60% and 90%, Figure 2).

We further estimated R_w_ and R_a_ for different sexes and age classes. Age classes were defined by pooling individuals aged 2–3, 4–5, 6–7, 8–9 and 10+ years, which provided adequate sample sizes within each class. Age-specific among-winter repeatability (R_a_) was estimated across each pair of consecutive winters separately, assigning individuals to their defined age class in the first winter. For example, estimates of R_a_ of individuals assigned to the 2–3 year age class used resightings from the winters of 2010–2011 and 2011–2012 of shags that hatched in 2008, resightings from all three winters of shags hatched in 2007, and resightings from winters 2009–2010 and 2010–2011 of shags hatched in 2006. Individuals ringed as chicks were of known age, and individuals ringed as adults were of estimated age: however, conclusions remained similar when analyses were restricted to individuals just ringed as chicks (Table S1).

The effect of age or sex on repeatability was tested using a variance partitioning approach [55]. Linear mixed effects models were fitted to the datasets describing the distances from the Isle of May of resightings for each winter with sex and age as fixed effects, and separate random sex and age effects [53], [56]. The fit of this model compared to models without random sex or age effects was quantified using likelihood ratio tests [56].

Analyses were implemented in R 2.15.0, and linear mixed effects models were fitted using the package lme4 [57], [58].

## Results

### Winter resighting data

A total of 313 positive survey days were distributed across all main survey sites in all months during all three winters (Figure S1), although the number of positive survey days varied among years (Table 1) and months (Figure S1). In total, 3797 resightings of 882 individual colour-ringed shags that were known to have bred on the Isle of May were recorded (Table 1). Of these, 561 individuals were resighted on ≥2 dates within one winter, and 449 were observed in ≥2 winters.

**Table 1:** Summary of the numbers of colour-ringed adult shags known to have bred on the Isle of May that were resighted during winters 2009–2012 across all survey sites.^1^

	Winter			
	2009–2010	2010–2011	2011–2012	Grand total
Total positive survey days	88	107	118	313
Median (IQR) positive survey days per month	13 (11–14)	14 (13–17)	20 (14–22)	
Median (IQR) positive survey sites per month	8 (7–11)	11 (10–14)	14 (11–15)	
Total resightings	700	1488	1609	3797
Total individuals resighted	317	575	605	882
Total individuals resighted on ≥2 dates	146	350	342	561
Median (IQR) resightings per individual resighted on ≥2 dates within a single winter	3 (2–4)	3 (2–4)	3 (2–4)	
Median (IQR) days between consecutive resightings of an individual resighted on ≥2 dates	16 (7–38)	18 (7–54)	12 (5–31)	
Median (IQR) days between first and last resightings of an individual resighted on >2 dates	73 (30–128)	106 (49–137)	76 (45–114)	

1 Grand totals are of unique individuals resighted across all winters, so do not equal the sum of individuals resighted within individual winters. Interquartile ranges (IQR) are shown in parentheses. Positive survey days are days on which at least one colour-ringed shag was resighted (Figure S1). Positive survey sites are roosts separated by ≥1 km where at least one colour-ringed shag was resighted on ≥1 date (Figure 1).

Overall, 42-66% of all colour-ringed adults observed breeding on the Isle of May in one summer were resighted during the following winter, and 19–41% were resighted on ≥2 dates within that winter (Table 2). Across individuals that were resighted on ≥2 dates, the median interval between the first and last sighting within a winter varied between 73 and 106 days across the three winters, and the median interval among consecutive resightings varied between 12–16 days (Table 1).

**Table 2:** Numbers of colour-ringed adult shags observed breeding on the Isle of May in each summer that were resighted during subsequent winters.^1^

			Summer		
			2009	2010	2011
Number of colour-ringed adults observed breeding	Grand Total		726	825	1010
		Male	383 (53%)	422 (52%)	499 (51%)
		Female	339 (47%)	391 (48%)	479 (49%)
Number of colour-ringed breeding adults resighted the subsequent winter	Total		303 (42%)	543 (66%)	561 (56%)
		Male	176 (58%)	279 (51%)	278 (50%)
		Female	127 (42%)	264 (49%)	283 (50%)
Number of colour-ringed breeding adults resighted on ≥2 dates the subsequent winter	Total		141 (19%)	338 (41%)	325 (32%)
		Male	97 (69%)	173 (51%)	164 (50%)
		Female	44 (31%)	165 (49%)	161 (50%)

1 Total numbers of colour-ringed adult shags with a recorded breeding attempt on the Isle of May in summers 2009, 2010 and 2011, and the total numbers resighted on ≥1 dates and ≥2 dates (and hence included in analyses of repeatability) during the subsequent winters. Total values in parentheses are the number of adults resighted in each winter expressed as a percentage of the grand total. Male and female values in parentheses are the number of each sex expressed as a percentage of the winter totals.

There was no systematic bias in the sexes or ages of individual shags that were resighted in winter compared to the observed Isle of May breeding population the preceding summer. Males comprised 50%–58% of all individuals resighted in the three winters, which did not differ significantly from the sex ratio of the previous summer's observed colour-ringed breeding population (Table 2, 2009–2010, χ^2^_1_ = 3.1, p = 0.08; 2010–2011, χ^2^_1_ = 0.1, p = 0.80; 2011–2012, χ^2^_1_ = 0.5, p = 0.49). Furthermore, the sex ratios of individuals resighted on ≥2 dates within a winter were similar, and did not differ significantly from those of the observed breeding population the previous summers, except that relatively more males were observed during the 2009–2010 winter (Table 2, 2009–2010, χ^2^_1_ = 14.0, p<0.01; 2010–2011, χ^2^_1_ = 0.1, p = 0.79; 2011–2012, χ^2^_1_<0.1, p = 0.84).

Individuals of a fully representative range of ages were resighted in winter relative to the ages of the observed Isle of May breeding population the previous summer (Figure S6). Neither the age distribution of all resighted individuals (2009–2010, χ^2^_13_ = 6.4, p = 0.93; 2010–2011, χ^2^_13_ = 3.9, p = 0.99; 2011–2012, χ^2^_13_ = 6.9, p = 0.91) or individuals resighted on ≥2 dates within a winter (2009–2010, χ^2^_13_ = 10.2, p = 0.68; 2010–2011, χ^2^_13_ = 9.5, p = 0.73; 2011–2012, χ^2^_13_ = 11.4, p = 0.58) differed significantly from that of the previous summer's observed breeding population.

### Total variation in resighting location

Colour-ringed adult shags that were known to have bred on the Isle of May were observed over a wide geographical range during the full winter periods 2009–2012, including on the Isle of May. At least one individual was resighted at all surveyed sites across the three winters, spanning 486 km north to 136 km south of the Isle of May (Figure 1).

The largest numbers of individuals were resighted on the Isle of May; the totals of 188 individuals in 2009–2010, 386 in 2010–2011 and 268 in 2011–2012 represent 37%, 49% and 33% of the total individuals resighted in each winter respectively (Table S3). Numerous individuals were resighted on the Isle of May even within the defined 50% movement boundaries within each winter: 77, 228 and 103 individuals in the 2009–2010, 2010–2011 and 2011–2012 winters respectively. Furthermore, including resightings from day roost locations adjacent to the Isle of May showed that many individuals remained in the Firth of Forth for at least part of the winter (totals of 236, 493 and 454 individuals were observed in the three winters respectively). Across the same three winter periods, 98, 124 and 173 individuals were resighted north of the Firth of Forth, while 4, 15 and 18 individuals were resighted south.

### Within-winter repeatability

In total, 146 colour-ringed adult shags that were known to have bred on the Isle of May were resighted on ≥2 dates across 15 different sites in the winter of 2009–2010, 350 across 21 sites in 2010–2011, and 342 across 21 sites in 2011–2012 (Table 3). These locations spanned total coastline distances of 512–724 km, and always included the Isle of May. Within each of the three full winter periods, the distance from the Isle of May at which individuals were resighted was highly and significantly repeatable within individuals (R_w_>0.72, Table 3).

**Table 3:** Within-winter repeatability (R_w_) of the distance from the Isle of May at which colour-ringed adult shags known to have bred on the Isle of May were resighted during winters 2009–2012.^1^

Winter	Movement boundary (%)	R_w_	V_i_	V_t_	p	No. individuals	No. resightings	No. sites	Distance range (km)
2009–2010	90	0.96	14055	14678	0.001	39	145	6	427
	60	0.88	15167	17264	0.001	92	343	10	427
	30	0.85	14450	16943	0.001	120	446	15	609
	0	0.80	12513	15721	0.001	146	529	15	609
2010–2011	90	0.95	19136	20164	0.001	74	260	13	512
	60	0.88	15251	17310	0.001	176	592	18	724
	30	0.74	9403	12685	0.001	322	1078	19	724
	0	0.72	8915	12411	0.001	350	1265	21	724
2011–2012	90	1.00	1190	1193	0.036	8	18	4	70
	60	0.90	14123	15575	0.001	138	439	16	410
	30	0.87	13518	15559	0.001	204	718	19	512
	0	0.85	12039	14191	0.001	342	1349	21	512

1 Repeatabilities were estimated across the whole winter period (1st September – 31st March, 0% movement boundary) and across increasingly restricted time periods defined by the 30%, 60% and 90% movement boundaries (Figure 2). Vi and Vt are the within-individual and total variances in distance from the Isle of May respectively, and p is the probability that the estimated Rw could be obtained by chance. The numbers of individuals, resightings and sites are the totals included in each analysis, with “sites” defined for descriptive purposes as the number of known roosts separated by ≥1 km. Distance range is the maximum coastline distance covered.

Within-winter repeatability of individual resighting distance was even higher across the subsets of resightings that fell within the restricted time periods defined by the movement boundaries (Table 3, Figure 2). This restriction inevitably reduced the sample size of individuals included (Table 3). However, at the 60% movement boundary, for example, R_w_ was very high (≥0.88) and higher than across the entire winter period (Table 3).

When within-winter repeatability was estimated for males and females separately, males had a higher R_w_ than females in all three winters (Table 4). This difference was marginally statistically significant in 2010–2011 (p = 0.05), but not in 2009–2010 (p = 0.91) or 2011–2012 (p = 0.56). The numbers of survey sites at which individuals were resighted and the total geographical ranges were broadly similar for both sexes. Within-winter repeatability did not differ consistently, or significantly, among age classes in any of the three winters (2009–2010, p = 0.79; 2010–2011, p = 0.15; 2010–2011, p = 0.97). However, R_w_ was always highest in the 6–7 year age class (0.81–1.00, Table 4).

**Table 4:** Within-winter repeatability (R_w_) of the distance from the Isle of May at which colour-ringed adults shags known to have bred on the Isle of May of different sexes and ages were resighted during winters 2009–2012.^1^

Winter	Sex/Age	R_w_	p	V_i_	V_t_	No. individuals	No. resightings	No. sites	Distance range (km)
2009–2010	Male	0.83	0.001	12796	15458	101	373	13	512
	Female	0.73	0.001	12053	16604	45	156	11	473
2010–2011	Male	0.78	0.001	10620	13586	180	730	18	512
	Female	0.60	0.001	6684	11083	167	518	20	676
2011–2012	Male	0.87	0.001	12873	14802	171	793	19	512
	Female	0.80	0.001	10614	13225	159	518	20	410
2009–2010	2–3	0.59	0.001	8901	15160	21	71	11	473
	4–5	0.79	0.001	11366	14467	90	327	20	410
	6–7	0.89	0.001	14640	16437	41	152	11	518
	8–9	0.86	0.001	14954	17458	27	110	7	291
	10+	0.69	0.001	12284	17767	22	87	6	291
2010–2011	2–3	0.69	0.001	9250	13457	61	221	12	376
	4–5	0.79	0.001	10055	12754	65	256	17	395
	6–7	0.81	0.001	12779	15853	26	85	9	291
	8–9	0.46	0.001	4705	10235	56	171	11	464
	10+	0.73	0.001	6917	9448	75	263	13	416
2011–2012	2–3	0.81	0.001	13045	16189	103	378	18	410
	4–5	0.79	0.001	8894	11224	89	343	19	724
	6–7	1.00	0.001	13302	13317	29	106	12	410
	8–9	0.89	0.001	11644	13023	55	271	16	434
	10+	0.86	0.001	11470	13389	80	307	12	408

1 Repeatabilities were estimated across the whole winter period (1^st^ September-31^st^ March, 0% movement boundary) for each year. V_i_ and V_t_ are the within-individual and total variances in distance from the Isle of May respectively, and p is the probability that the estimated R_w_ could be obtained by chance. The numbers of individuals, resightings and sites are the totals included in each analysis, with “sites” defined for descriptive purposes as the number of roosts separated by ≥1 km. Distance range is the maximum coastline distance covered. Ages are years since hatching at the start of each winter. The analysis includes individuals ringed as adults and therefore of estimated age: for individuals ringed as chicks only, see Table S1.

### Among-winter repeatability

Overall, 449 colour-ringed adult shags that were known to have bred on the Isle of May were resighted in ≥2 winters, encompassing 29 sites spanning a total coastline distance of 512 km and including the Isle of May (Table 5). Across all three full winter periods, the distance from the Isle of May at which individuals were resighted was highly repeatable (R_a_ = 0.59, Table 5). As with R_w_, R_a_ was even higher when estimated across increasingly restricted movement boundaries (e.g. R_a_ = 0.73 at the 60% movement boundary, Figure 2). The sampling variance generated from repeated random selection of single resightings per individual per winter was small (Table 5). Individual shags therefore show high winter site fidelity among winters as well as within winters, although R_a_ was lower than R_w_ across the full winter period and across each movement boundary (Table 6).

**Table 5:** Among-winter repeatability (R_a_) of the distance from the Isle of May at which colour-ringed adult shags known to have bred on the Isle of May were resighted during winters 2009–2012.^1^

Movement boundary (%)	R_a_	V_i_	V_t_	p	Sampling interval	No. individuals	No. resightings	No. sites	Distance range (km)
90	0.79	14502	18357	0.001	0.77–0.79	74	164	15	512
60	0.73	10948	15077	0.001	0.73–0.73	242	558	22	512
30	0.64	7963	12528	0.001	0.63–0.64	360	841	27	512
0	0.59	6781	11439	0.001	0.59–0.59	449	1064	29	512

1 Repeatabilities were estimated across the whole winter period (1st September – 31st March, 0% movement boundary) and across increasingly restricted time periods defined by the 30%, 60% and 90% movement boundaries (Figure 2). Vi and Vt are the within-individual and total variances in the distance from the Isle of May respectively, and p is the probability that the estimated Ra could be obtained by chance. The sampling interval is the upper and lower 95% intervals of the Ra estimates obtained by resampling a single resighting of each individual within each winter. The numbers of individuals, resightings and sites are the totals included in each analysis, with “sites” defined for descriptive purposes as the number of roosts separated by ≥1 km. Distance range is the maximum coastline distance covered.

**Table 6:** Among winter repeatability (R_a_) of the distance from the Isle of May at which colour-ringed adult shags known to have bred on the Isle of May of different sexes and ages were resighted during winters 2009–2012.^1^

Sex/Age	R_a_	p	V_i_	V_t_	No. individuals	No. resightings	No. sites	Distance range (km)
**Male**	0.64	0.001	7705	12064	237	578	24	512
**Female**	0.53	0.001	5635	10699	210	481	21	464
**2–4**	0.39	0.001	5281	13471	89	174	14	399
**4–6**	0.56	0.001	5531	9926	113	234	22	410
**6–8**	0.76	0.001	8665	11373	128	279	21	512
**8–10**	0.53	0.001	5803	10945	81	171	16	464
**10+**	0.41	0.001	6292	15408	91	206	13	410

1 Repeatabilities were estimated across the whole winter period (1^st^ September – 31^st^ March, 0% movement boundary). V_i_ and V_t_ are the within-individual and total variances of distance from the Isle of May respectively, and p is the probability that the estimated R_a_ could be obtained by chance. The numbers of individuals, resightings and sites are the totals included in each analysis, with “sites” defined for descriptive purposes as the number of roosts separated by ≥1 km. Distance range is the maximum coastline distance covered. Age is the number of years since hatching at the start of each winter. Ages are listed by the age transitions among winters included in the analysis, i.e. 2–3 includes a resighting of all individuals aged 2 in any winter which were also resighted in the subsequent winter when they were aged 3. The analysis includes individuals ringed as adults and therefore of estimated age: for individuals ringed as chicks only, see Table S1.

Across all winters, R_a_ was higher for males than females, but this difference was not significant (p = 0.33). Overall, R_a_ did not differ significantly among age-classes (p = 0.10). However, estimates were high for 6–8 year old shags, and lower for 2–4 and 10+ year olds (Table 6).

## Discussion

Quantifying the degree to which individual population members differ consistently in migration strategies and locations, and therefore systematically experience different environments within and among seasons and years, is prerequisite to understanding life-history variation and consequent population dynamics [12], [13], [59]. Using structured surveys and *ad hoc* resightings, we resighted 882 colour-ringed adult European shags that were known to have bred on the Isle of May, an internationally important breeding colony, across 622 km of coastline during three winters. Substantial among-individual variation yet very high within-individual repeatability in winter location was evident, both within and among winters. These data demonstrate that the population is partially migratory with different resident and migrant individuals consistently experiencing geographically distinct environments, potentially creating structured variation in life-history.

Our aim was to repeatedly resight individuals across a sample of locations over a large geographical range, not to map the winter distribution of all individuals known to have bred on the Isle of May. A substantial proportion of the population consequently wintered in unknown locations, probably including small, scattered or unobservable locations such as sea caves within the surveyed geographical region. However, colour-ring resightings provided geographically accurate winter locations of over 50% of the known breeding population (Table 2). Furthermore, the sex and age distributions of colour-ringed shags resighted in winter did not differ from those of the known breeding population, demonstrating that representative subsets of individuals were resighted (Figure S6). Field resightings can therefore provide sparse but large-scale locational data on a large number of individuals within and among seasons and years, potentially complementing logger data that can provide higher frequency fixes, but often for more restricted subsets of individuals [29], [60], [61]. In their seminal study, Gunnarsson *et al*. [62] resighted over 50% of colour-ringed individual black-tailed godwits (*Limosa limosa*) on both wintering and breeding grounds, but only 1–2% of the estimated breeding population was marked. One strength of the shag system is therefore that a substantial and representative proportion of an internationally important breeding colony can be located across the full likely extent of its geographical range in winter (see also [42]).

### Within-winter repeatability

Colour-ringed adult shags known to have bred on the Isle of May were observed from 486 km north to 136 km south of the Isle of May in winter, demonstrating substantial variation in winter location (Figure 1). The distances from the Isle of May at which individual shags were resighted were highly repeatable within winters. Estimated repeatabilities were high across the entire September to March survey period (R_w_≥0.72), and even higher across increasingly restricted mid-winter periods (e.g., R_w_≥0.95 within the 90% movement boundary, when 90% of those individuals observed to have migrated were away from the Isle of May, Figure 2, Table 3). However, some within-individual variation within each movement boundary undoubtedly still reflects migration between the Isle of May and other winter locations, suggested by R_w_ approaching 1 as the winter period was increasingly restricted (Table 4). Similarly, Frederiksen *et al*. [63] used colour-ring resightings to investigate site fidelity in cormorants, and concluded that considering a wider “winter” period (November-February rather than December-February) led to lower estimates of fidelity.

Even given an increasingly restricted winter period, R_w_ was generally less than 1, showing that some within-individual variation in resighting distance remained (Table 3). Some of this variation arises because many individuals were resighted at ≥2 sites within commuting distance of each other, such as adjacent night and day roosts or alternative day roosts used under different weather and tidal conditions. In particular, numerous individuals were resighted at three day roosts located ≤14 km away from the Isle of May night roost (sites 19, 20 & 21, Figure 1). Overall, the median distance between resightings for individuals resighted at ≥2 sites was only 7 km both within and among winters (Figure S2). Hobson & Sealy [64] found a similar pattern of site use in pelagic cormorants (*Phalacrocorax pelagicus*) in British Columbia, where individuals irregularly used a range of daytime roost sites between foraging bouts. Part of the within-individual variation in resighting distance therefore reflects hour to hour use of multiple close sites which can be considered a single biologically relevant location, rather than longer movements among different geographical locations.

A further potential explanation for the remaining within-individual variation is that some individuals may be following different behavioural strategies; for example, individuals that migrated away from the Isle of May might be more mobile throughout the winter. However, further analysis of all resightings of individuals that had ever been resighted at three key night roosts spread over 291 km (sites 5, 11 and Isle of May, Figure 1) suggests that this was not the case. Specifically, individual shags that were known to have bred on the Isle of May and resighted at a different winter night roost were rarely resighted anywhere else within the same winter, although more individuals that were resighted on the Isle of May were also resighted elsewhere, as expected given migration (Figure S7).

Survey effort spanned all main sites and all months across all three winters (Figure S1), although the exact distribution of positive survey days varied with season and weather conditions, and mid-winter surveys of the Isle of May were less frequent due to seasonal inaccessibility. However, despite the broad temporal distribution of surveys, estimates of R_w_ could potentially be upwardly biased by resightings of individuals that are close in time and hence potentially autocorrelated in space, or biased by pre- or post-migration resightings in early or late winter that may not describe an individual's main winter location. However, the distribution of the intervals between the first and last resightings of an individual (Figure S4) was not heavily skewed towards extremely long or short durations. Estimates of R_w_ remained similar when analyses were restricted to individuals where the interval between their first and last resightings fell within the population-wide interquartile range (Table S2).

Since analyses of R_w_ were restricted to individuals that were resighted on ≥2 dates within a single winter, estimates could potentially be upwardly biased if this restriction excluded individuals with low site fidelity and consequently lower probability of being resighted multiple times at the survey sites. Of all 882 individuals that were resighted during the 2009–2012 winters, 321 (36%) were only resighted once within any single winter (although some were resighted in multiple winters). However, many surveyed sites varied daily, annually, tidally and in different weather conditions in patterns of use, and are also only partially visible from land. The probability of resighting a colour-ringed shag that was present can consequently be considerably <1. In addition, some resightings came from sites that were surveyed infrequently, meaning that individuals that showed high fidelity to these sites were unlikely to be repeatedly resighted. Of those individuals resighted only once, a substantial proportion (44%) were resighted on the Isle of May (Figure S3). These likely comprise pre- and post-migration resightings of individuals that migrated to unsurveyed sites, and the less frequent mid-winter surveys on the Isle of May which result in a lower probability of resighting resident individuals. Excluding individuals that were resighted once (or never) is therefore unlikely to invalidate the overall inference that individual shags typically show extremely high site fidelity within winters.

As expected given the high overall R_w_, values of R_w_ did not differ significantly between males and females or among age-classes. However, estimates of R_w_ were consistently slightly higher for males than females, perhaps reflecting male winter territorial dominance [65], [66]. Estimates of R_w_ varied more among age classes than between the sexes; shags aged 6–7 consistently had higher R_w_ than younger or older individuals. This could again potentially reflect winter dominance; middle-aged shags have intrinsically higher breeding success and lower mortality linked to better foraging performance [67], and are behaviourally dominant [8]. These patterns remained similar when analyses were restricted to individuals that had been ringed as chicks, therefore eliminating uncertainty in the ages of individuals ringed as adults (Table S1).

### Among-winter repeatability

Shags known to have bred on the Isle of May were also highly repeatable in the distance from the Isle of May at which they were resighted across different winters. Estimated R_a_ was 0.59 across the full September-March survey periods, and ≥0.79 within the restricted mid-winter period when 90% of observed migrants were estimated to be away from the Isle of May (Figure 2, Table 5).

Although R_a_ was high, it was <1 and also lower than R_w_. Some within-individual variation in R_a_ is likely to arise because individuals were observed before and after migrating in different winters, rather than necessarily because those individuals changed their wintering location. The distances between the sites at which individuals were resighted across winters were similar to those within winters, and largely reflect use of several adjacent sites, or migration (Figure S2). Again, individuals resighted at key winter night roosts (sites 5, 11 and 22, Figure 1) were rarely resighted elsewhere in subsequent winters (Figure S7). Although 432 (49%) of all 882 resighted adult shags were only seen in one winter, the highest proportions of individuals resighted in only one winter were also on the Isle of May (Figure S3). In addition, a proportion of individuals resighted in only one winter were likely to have died between years, or were only classed as a known Isle of May breeding adult in one winter and so were not available for resightings across more years.

These data imply that both resident and migratory shags that breed on the Isle of May show high site fidelity across winters, meaning that individuals follow consistent migratory strategies. Previous work suggests that individual migratory strategies within partially migratory populations can be genetically determined and therefore obligate, or condition-dependent and flexible [68]–[70]. Whether migratory strategy is largely genetically or environmentally determined will influence both individual and population dynamic responses to environmental change. The high R_a_ values estimated in shags may indicate obligate partial migration; however, our study only encompassed three winters, and during 2009–2012 annual return rates for adult shags breeding on the Isle of May were very high (0.93–0.95) suggesting that environmental conditions were consistently good. Frederiksen *et al*. [63] used colour-ring resightings to quantify site fidelity in cormorants during 1980–2002, and showed that although only 10–15% of individuals changed site among years, these changes were likely due to environmental changes or disturbance. Further years of data, spanning a greater diversity of winter conditions, are therefore needed to determine if shag migratory strategies and site fidelity remain consistent over longer time scales with greater variability in environmental conditions.

In addition, there was no relationship between repeatability and age, which suggests that adult shags of all ages show high site fidelity. However, the current cross-sectional analysis does not allow further investigation of changes in site fidelity or migratory strategy over individual lifespans, or of how and when an individual's migratory strategy is determined. Ringing recoveries suggest that juvenile shags move further from the breeding colony during winter than adults, and individual movements during the period between fledging and recruitment may therefore be important in determining adult winter location [34], [71]. Quantifying the movements of juveniles during the pre-recruitment phase and the relationship with subsequent migratory strategy is therefore an important future stage in understanding the causes of individual variation in winter location, and the subsequent effects on population dynamics.

### Conclusions and implications

Our data demonstrate high among-individual variation in the winter locations of adult shags known to have bred in a single colony, coupled with high individual site fidelity both within and among winters. The population is therefore partially migratory, with consistently migratory individuals occupying consistent locations within a large geographical range. Although previous data indicate that some shags breeding on the Isle of May do leave the colony in winter [42], [47], [48], the current analyses provide the first explicit evidence of partial migration. Many species of Phalacrocoracidae are resident or only disperse short distances, except under severe winter conditions. However, a recent satellite telemetry study on pelagic cormorants (*Phalacrocorax pelagicus*) showed that individuals migrate an average of 920 km from the breeding colony [61], and ring recoveries suggest that European shags breeding in Croatia have increased winter movements over a ca. 30 year period to become fully migratory [71]. Our study highlights that quantifying individual variation in winter distribution within a single population can reveal previously unknown movements of individuals within a large geographical range.

Although partial migration is found across a range of taxa and geographical areas [69], [70], [72], there is limited evidence in other seabirds. However, recent work has demonstrated a previously unknown division in the winter movements of black-legged kittiwakes (*Rissa tridactyla*) among individuals that remained resident and those that migrated up to 1700 km offshore [73], and common diving petrels (*Pelecanoides urinatrix*) were shown to occupy a diverse range of coastal and pelagic winter locations [74]. In addition, non-breeding season site fidelity has been demonstrated in a range of species, including birds and cetaceans [21], [75]. However, it is by no means universal: lone grizzly bears (*Ursa arctos*) are transient across a large landscape [76], and only 26% of a single population of blackcaps (*Sylvia atricapilla*) were found to show site fidelity [77].

Understanding the reliance of individuals on specific sites is key to implementing effective conservation management, as populations can be sensitive to environmental change at these sites [78]. White-fronted geese (*Anser albifrons*) showed high site fidelity even when habitat conditions deteriorated [34] and populations of harlequin ducks (*Histrionicus histrionicus*) with high fidelity to areas affected by the Exxon Valdez Oil spill in Alaska were slow to recover, which was attributed to strong spatial structure and subsequent slow colonisation of available sites [79]. Shags that breed on the Isle of May are protected under national and international legislation, but our study shows that a substantial proportion of the population show strong site fidelity to a range of different winter locations, suggesting that current protection may be insufficient.

In general, variation in winter location and site fidelity can substantially affect over-winter survival within single breeding populations of a range of species [77], [80], and have longer-term carry-over effects into following seasons [10], [81]. Population-wide survival of adult shags has previously been shown to be influenced by environmental variation during the winter [43], and timing of breeding has been linked to winter foraging [47]. The consistent high among-individual variation and low within-individual variation in winter locations of shags breeding on the Isle of May means that individuals consistently experience different winter environments, and may consequently be an important source of demographic heterogeneity, with potentially fundamental impacts on population dynamics.

## Supporting Information

Figure S1 **Positive survey days for colour-ringed shags per month.** The total number of positive survey days per month during 1^st^ September – 31^st^ March 2009–2010 (dashed line), 2010–2011 (black line), and 2011–2012 (grey line). Positive survey days are defined as dates where ≥1 colour-ringed adult shag known to have bred on the Isle of May was resighted at ≥1 site.
(TIF)

Figure S2 **Distance between consecutive resightings of individual colour-ringed shags within and among winters.** The distance (km) between consecutive resightings for individual colour-ringed adult shags known to have bred on the Isle of May that were resighted at ≥2 sites during 1^st^ September- 31^st^ March in A. a single winter 2009–2010, 2010–2011 and 2011–2012 and B. among all three winters.
(TIF)

Figure S3 **Resighting sites of colour-ringed shags that were resighted only once within and among winters.** Resighting site of colour-ringed adult shags known to have bred on the Isle of May that were only resighted once (light grey) compared to the total numbers of individuals resighted at those sites (dark grey) during winters A. 2009–2010, B. 2010–2011, C. 2011–2012 and D. among all three winters.
(TIF)

Figure S4 **Intervals between first and last resightings of individual colour-ringed shags within winters.** The number of days between the first and last resightings of colour-ringed adult shags known to have bred on the Isle of May that were resighted on ≥2 dates during 1^st^ September – 31^st^ March in A. 2009–2010, B. 2010–2011 and C. 2011–2012. Dashed lines indicate the interquartile range.
(TIF)

Figure S5 **Intervals between consecutive resightings of individual colour-ringed shags within winters.** The distribution of the number of days between all pairs of consecutive resightings of individual colour-ringed adult shags known to have bred on the Isle of May that were resighted on ≥2 dates during 1^st^ September- 31^st^ March in A. 2009–2010, B. 2010–2011 and C. 2011–2012.
(TIF)

Figure S6 **Age distributions of shags known to have bred on the Isle of May and resighted the following winter.** Proportional age distribution of colour-ringed adult shags relative to the total numbers observed breeding on the Isle of May (black bars), individuals resighted across all survey locations during 1^st^ September-31^st^ March the subsequent winter (white bars), and individuals resighted on ≥2 dates across the same period (grey bars), relative to the A. 2009, B. 2010, and C. 2011 breeding seasons.
(TIF)

Figure S7 **All resightings of colour-ringed shags at three focal night roost sites.** Distances from the Isle of May of all resightings of colour-ringed adult shags known to have bred on the Isle of May that were resighted on ≥2 dates within a winter or in ≥2 winters at site 5 (white), site 11 (grey) or Isle of May (black) in winters A. 2009–2010, B. 2010–2011, C. 2011–2012 and D. among winters (Figure 1). Y axis shows distance from the Isle of May in km. Bars are overlaid rather than stacked.
(TIF)

Table S1 **Within- and among- winter repeatability of distance from the Isle of May at which colour-ringed adult shags, ringed as chicks on the Isle of May and known to have bred there as adults, were resighted during winters 2009–2010, 2010–2011 and 2011–2012.**
(PDF)

Table S2 **Within- and among- winter repeatability (R) of distance from the Isle of May at which adult colour-ringed shags known to have bred on the Isle of May, and with an interval between first and last resightings that falls within the population-wide interquartile range, were resighted during winters 2009–2012.**
(PDF)

Table S3 **Numbers of individual colour-ringed adult shags that were known to have bred on the Isle of May that were resighted at each winter survey site during winters 2009–2010, 2010–2011 and 2011–2012.**
(PDF)
