# Supplementary figures and images for: Site Fidelity and Individual Variation in Winter Location in Partially Migratory European Shags
Source: PLoS One. 2014 Jun 3;9(6):e98562. doi: 10.1371/journal.pone.0098562 (PMC4043777; doi:10.1371/journal.pone.0098562)

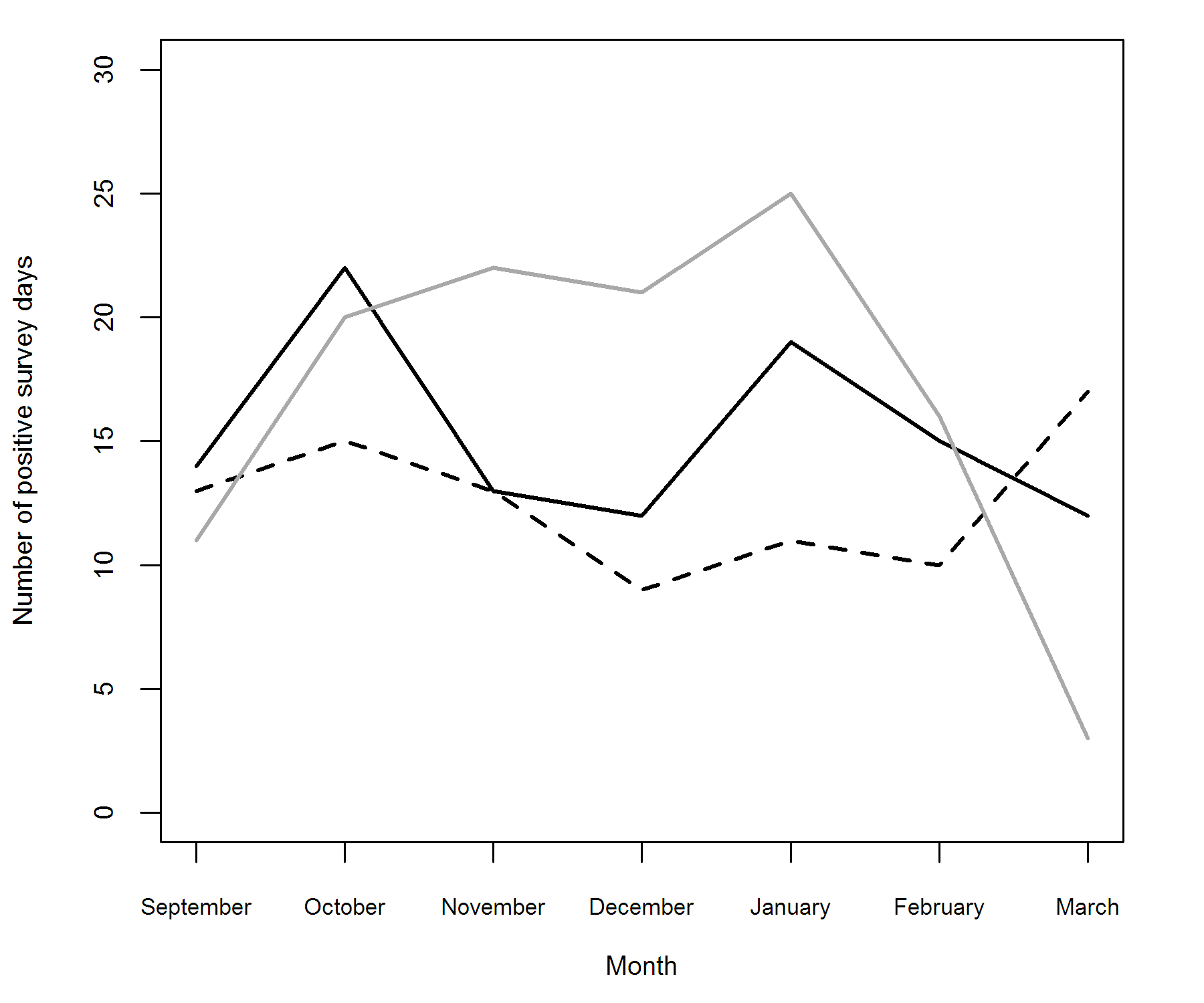

Supplement: Figure S1 — Positive survey days for colour-ringed shags per month. The total number of positive survey days per month during 1st September – 31st March 2009–2010 (dashed line), 2010–2011 (black line), and 2011–2012 (grey line). Positive survey days are defined as dates where ≥1 colour-ringed adult shag known to have bred on the Isle of May was resighted at ≥1 site. (TIF) [file pone.0098562.s001.tif]

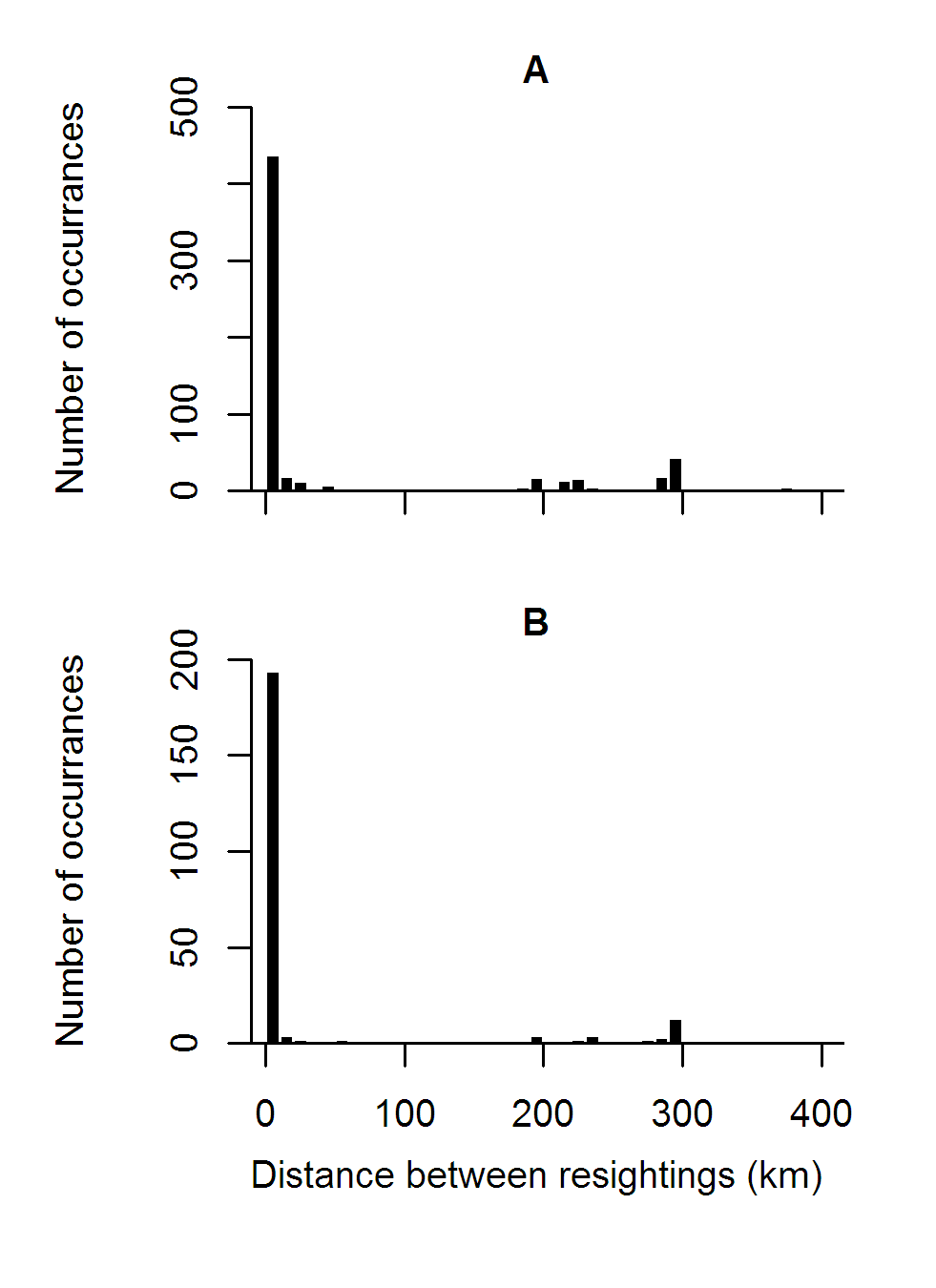

Supplement: Figure S2 — Distance between consecutive resightings of individual colour-ringed shags within and among winters. The distance (km) between consecutive resightings for individual colour-ringed adult shags known to have bred on the Isle of May that were resighted at ≥2 sites during 1st September- 31st March in A. a single winter 2009–2010, 2010–2011 and 2011–2012 and B. among all three winters. (TIF) [file pone.0098562.s002.tif]

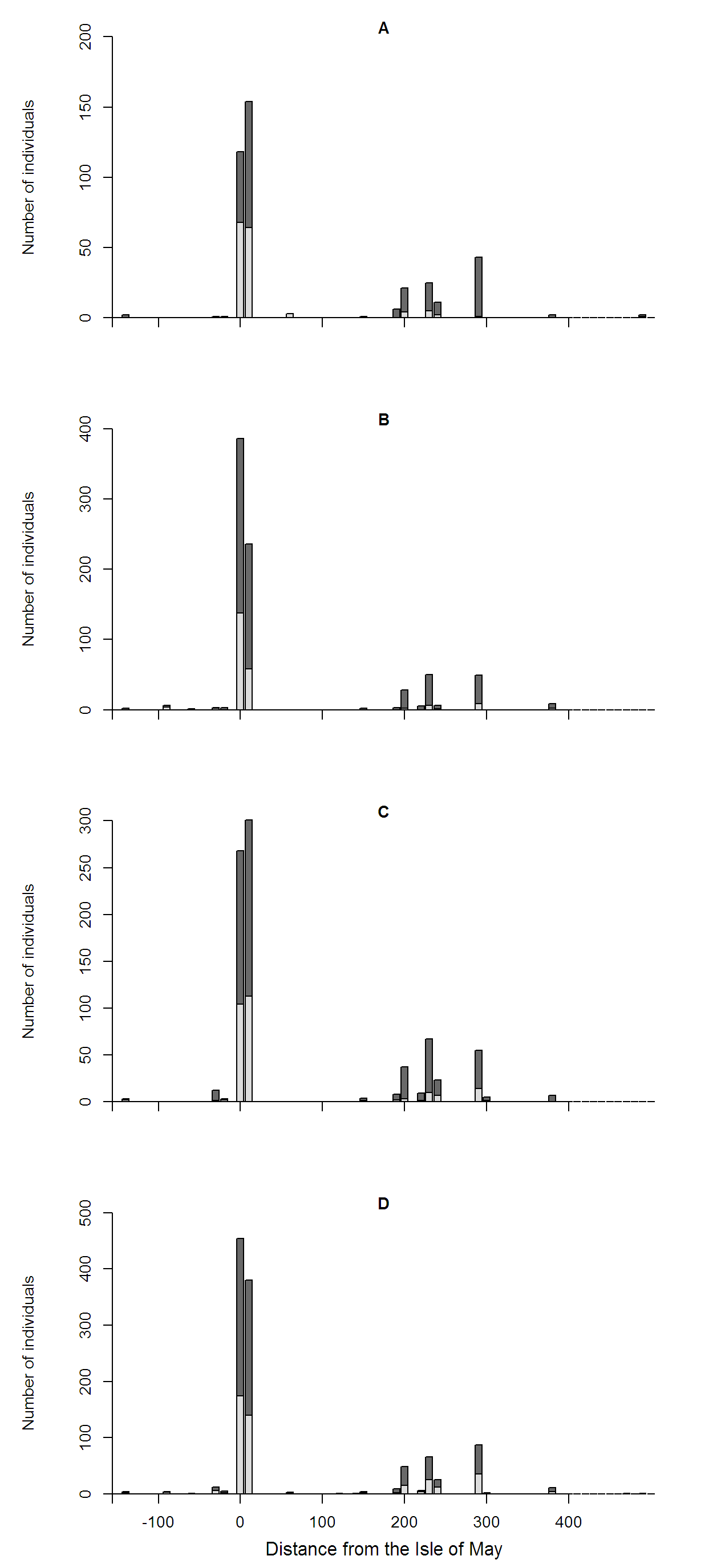

Supplement: Figure S3 — Resighting sites of colour-ringed shags that were resighted only once within and among winters. Resighting site of colour-ringed adult shags known to have bred on the Isle of May that were only resighted once (light grey) compared to the total numbers of individuals resighted at those sites (dark grey) during winters A. 2009–2010, B. 2010–2011, C. 2011–2012 and D. among all three winters. (TIF) [file pone.0098562.s003.tif]

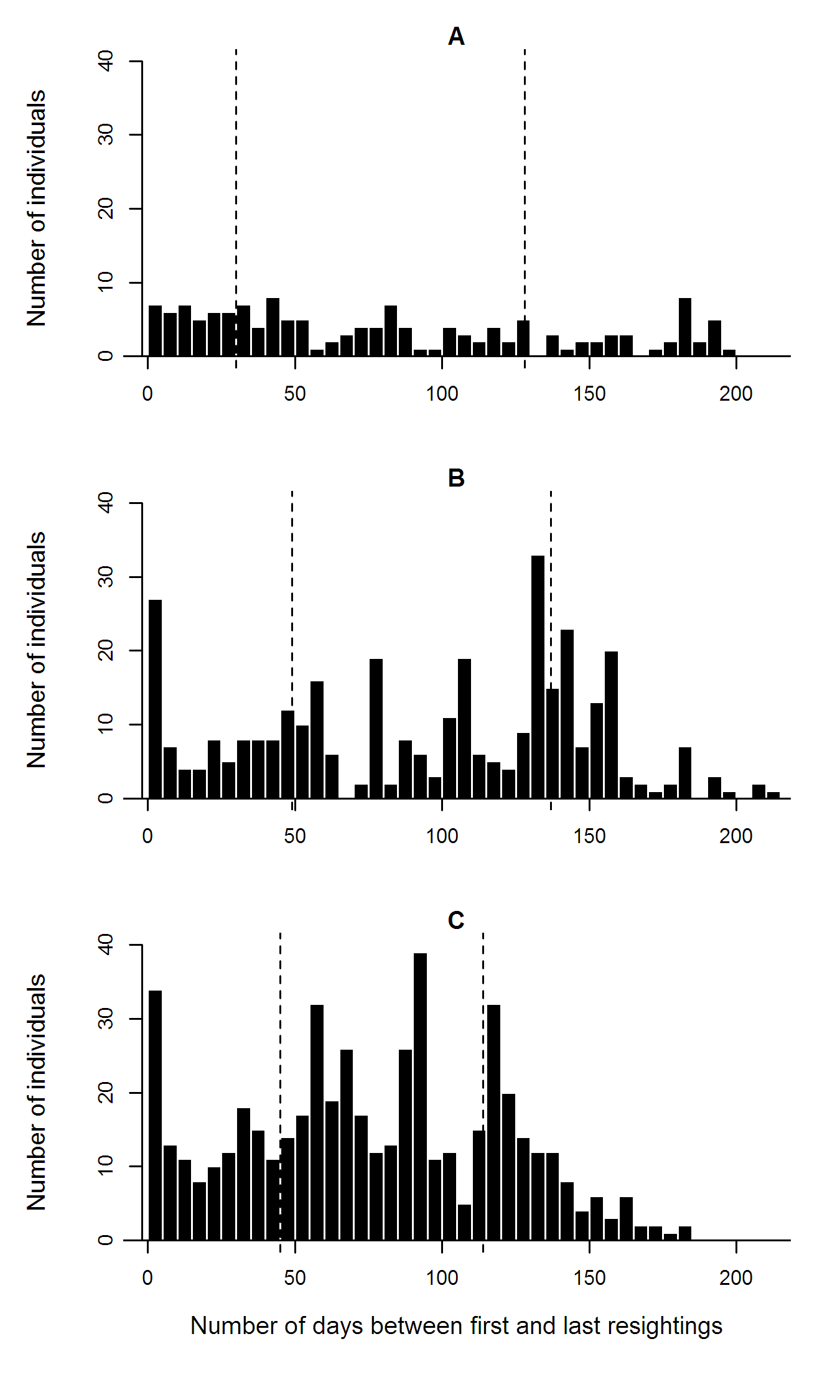

Supplement: Figure S4 — Intervals between first and last resightings of individual colour-ringed shags within winters. The number of days between the first and last resightings of colour-ringed adult shags known to have bred on the Isle of May that were resighted on ≥2 dates during 1st September – 31st March in A. 2009–2010, B. 2010–2011 and C. 2011–2012. Dashed lines indicate the interquartile range. (TIF) [file pone.0098562.s004.tif]

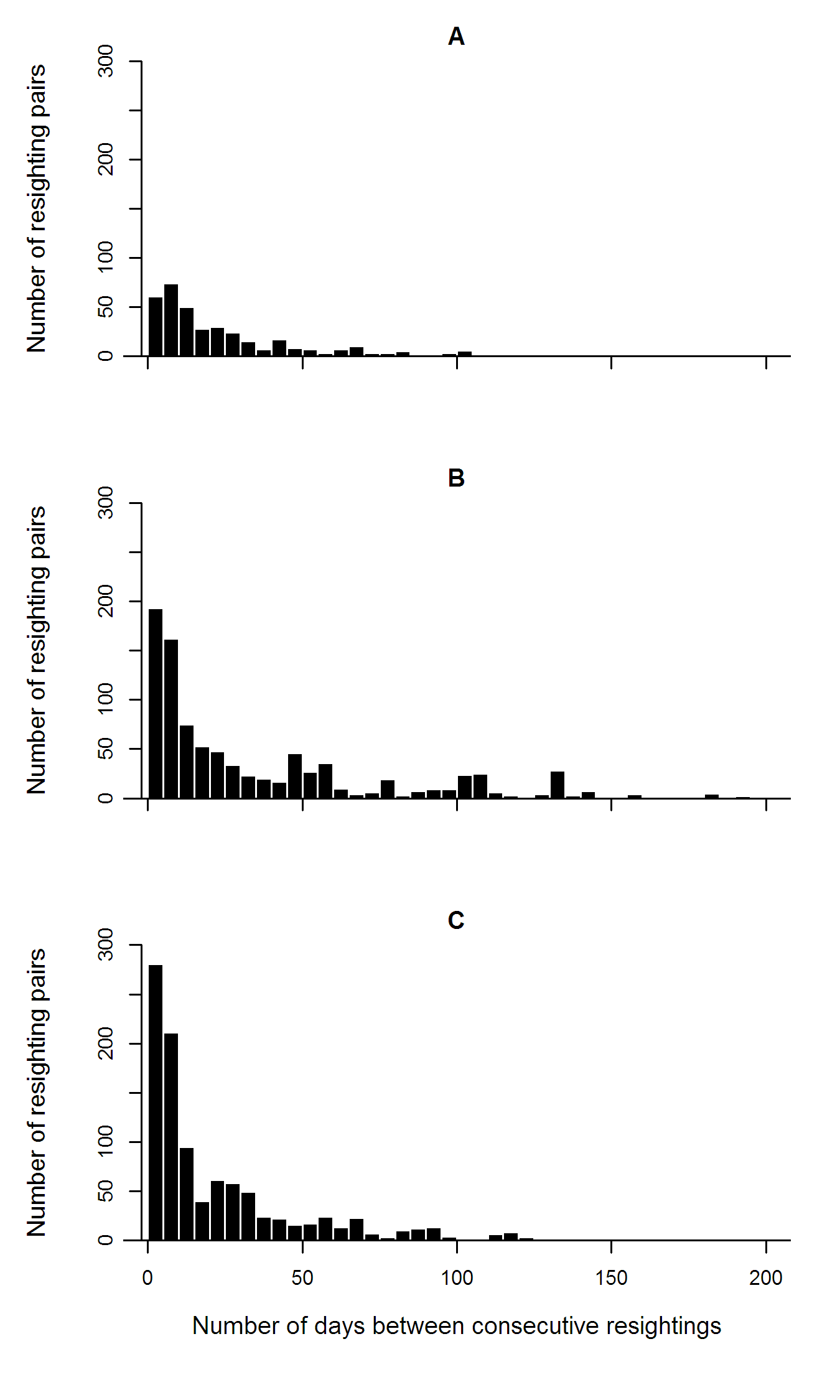

Supplement: Figure S5 — Intervals between consecutive resightings of individual colour-ringed shags within winters. The distribution of the number of days between all pairs of consecutive resightings of individual colour-ringed adult shags known to have bred on the Isle of May that were resighted on ≥2 dates during 1st September- 31st March in A. 2009–2010, B. 2010–2011 and C. 2011–2012. (TIF) [file pone.0098562.s005.tif]

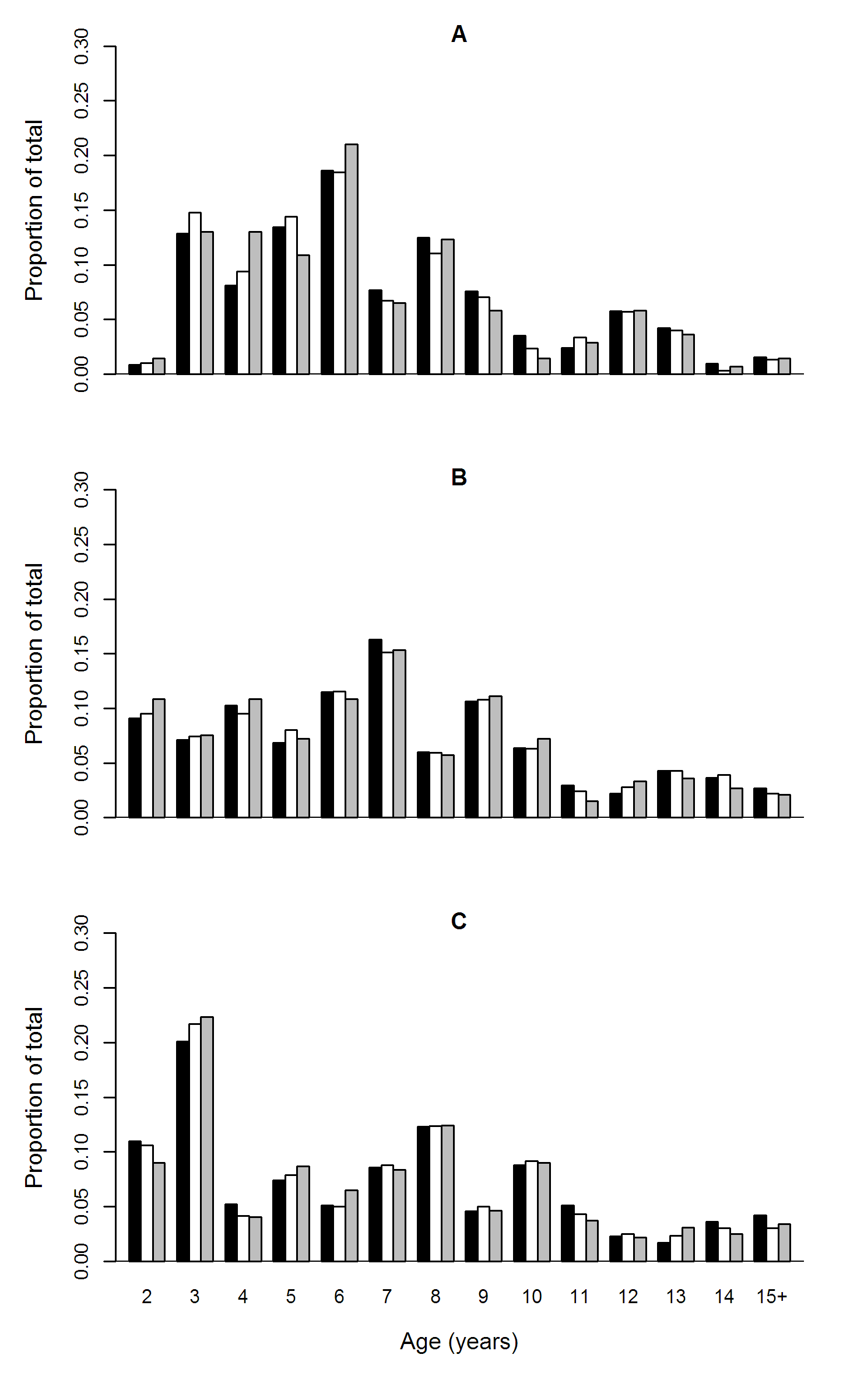

Supplement: Figure S6 — Age distributions of shags known to have bred on the Isle of May and resighted the following winter. Proportional age distribution of colour-ringed adult shags relative to the total numbers observed breeding on the Isle of May (black bars), individuals resighted across all survey locations during 1st September-31st March the subsequent winter (white bars), and individuals resighted on ≥2 dates across the same period (grey bars), relative to the A. 2009, B. 2010, and C. 2011 breeding seasons. (TIF) [file pone.0098562.s006.tif]

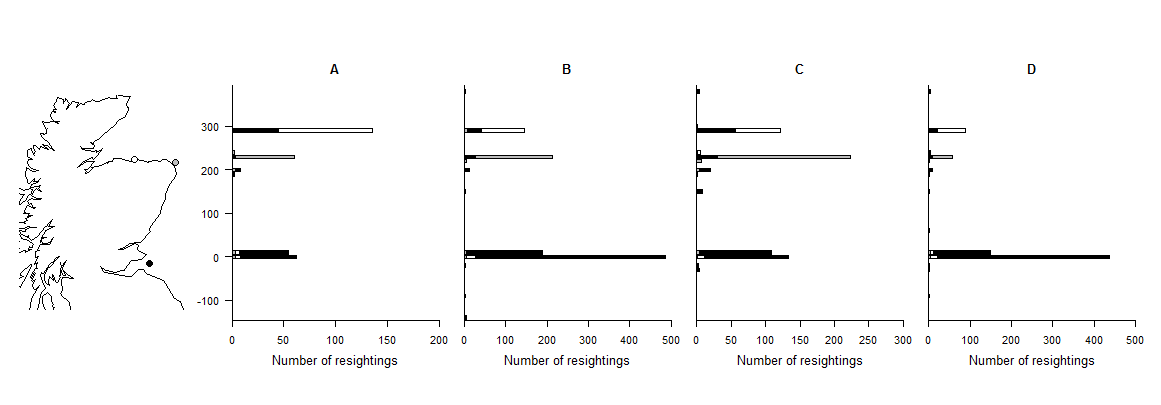

Supplement: Figure S7 — All resightings of colour-ringed shags at three focal night roost sites. Distances from the Isle of May of all resightings of colour-ringed adult shags known to have bred on the Isle of May that were resighted on ≥2 dates within a winter or in ≥2 winters at site 5 (white), site 11 (grey) or Isle of May (black) in winters A. 2009–2010, B. 2010–2011, C. 2011–2012 and D. among winters (Figure 1). Y axis shows distance from the Isle of May in km. Bars are overlaid rather than stacked. (TIF) [file pone.0098562.s007.tif]
