# Supplementary material for: Site Fidelity and Individual Variation in Winter Location in Partially Migratory European Shags
Source: PLoS One. 2014 Jun 3;9(6):e98562. doi: 10.1371/journal.pone.0098562 (PMC4043777; doi:10.1371/journal.pone.0098562)
Supplement: Table S1 — Within- and among- winter repeatability of distance from the Isle of May at which colour-ringed adult shags, ringed as chicks on the Isle of May and known to have bred there as adults, were resighted during winters 2009–2010, 2010–2011 and 2011–2012. (PDF) [file pone.0098562.s008.pdf]

**Table S1. Within- and among- winter repeatability of distance from the Isle of May at which colour-ringed adult shags, ringed as chicks on the Isle of May and known to have bred there as adults, were resighted during winters 2009-2010, 2010-2011 and 2011-2012.**

| Winter | Age/Sex | $R_{all}$ | $R_{chicks}$ | p | $V_i$ | $V_t$ | No. individuals | No. resightings | No. sites | Distance range (km) |
|---|---|---|---|---|---|---|---|---|---|---|
| **2009-2010** | 2-3 | 0.59 | 0.59 | 0.001 | 11075 | 18915 | 9 | 27 | 5 | 380 |
| | 4-5 | 0.79 | 0.74 | 0.001 | 10245 | 13778 | 54 | 222 | 17 | 408 |
| | 6-7 | 0.89 | 0.92 | 0.001 | 18088 | 19737 | 27 | 106 | 10 | 518 |
| | 8-9 | 0.86 | 0.85 | 0.001 | 15553 | 18403 | 23 | 96 | 7 | 291 |
| | 10+ | 0.69 | 0.67 | 0.001 | 12371 | 18523 | 19 | 77 | 6 | 291 |
| **2010-2011** | 2-3 | 0.69 | 0.81 | 0.001 | 14676 | 18093 | 43 | 167 | 11 | 376 |
| | 4-5 | 0.79 | 0.75 | 0.001 | 9968 | 13279 | 19 | 87 | 10 | 310 |
| | 6-7 | 0.81 | 0.88 | 0.001 | 12656 | 14375 | 3 | 9 | 4 | 291 |
| | 8-9 | 0.46 | 0.44 | 0.001 | 5268 | 11877 | 47 | 143 | 10 | 464 |
| | 10+ | 0.73 | 0.73 | 0.001 | 6979 | 9584 | 67 | 234 | 12 | 416 |
| **2011-2012** | 2-3 | 0.81 | 0.80 | 0.001 | 13295 | 16524 | 80 | 304 | 18 | 410 |
| | 4-5 | 0.79 | 0.68 | 0.001 | 9441 | 13888 | 42 | 189 | 16 | 427 |
| | 6-7 | 1.00 | 0.88 | 0.001 | 12656 | 14375 | 3 | 21 | 1 | 410 |
| | 8-9 | 0.89 | 0.89 | 0.001 | 14183 | 15906 | 38 | 211 | 15 | 434 |
| | 10+ | 0.86 | 0.85 | 0.001 | 11605 | 13666 | 73 | 284 | 11 | 408 |
| **Among winters** | 2-3 | 0.39 | 0.45 | 0.001 | 6719 | 14852 | 54 | 110 | 15 | 395 |
| | 4-5 | 0.56 | 0.47 | 0.001 | 6809 | 14528 | 22 | 45 | 10 | 330 |
| | 6-7 | 0.76 | 0.80 | 0.001 | 11252 | 14042 | 63 | 147 | 19 | 427 |
| | 8-9 | 0.53 | 0.53 | 0.001 | 6657 | 12562 | 69 | 152 | 16 | 464 |
| | 10+ | 0.41 | 0.40 | 0.001 | 6533 | 16432 | 82 | 192 | 12 | 464 |

Repeatabilities were estimated across the whole of each winter period (1$^{st}$ September- 31$^{st}$ March) for each year for individuals ringed as chicks and therefore of exact known age ($R_{chicks}$). $V_i$ and $V_t$ are the within-individual and total variances of resighting distance from the Isle of May respectively, and p values are the probability of the estimated $R_{chick}$ values occuring by chance. The numbers of individuals, resightings and sites are the totals included in each analysis, with “sites” defined for descriptive purposes as the number of known roosts separated by ≥1km. Distance range is the maximum coastline distance covered. Ages are years since hatching at the beginning of the winter of individuals included in the analysis. Repeatabilities calculated across all resighted individuals including those ringed as adults and hence of estimated age are also shown for comparative purposes ($R_{all}$, see also Table 4).
