# Supplementary material for: Site Fidelity and Individual Variation in Winter Location in Partially Migratory European Shags
Source: PLoS One. 2014 Jun 3;9(6):e98562. doi: 10.1371/journal.pone.0098562 (PMC4043777; doi:10.1371/journal.pone.0098562)
Supplement: Table S2 — Within- and among- winter repeatability (R) of distance from the Isle of May at which adult colour-ringed shags known to have bred on the Isle of May, and with an interval between first and last resightings that falls within the population-wide interquartile range, were resighted during winters 2009–2012. (PDF) [file pone.0098562.s009.pdf]

Table S2. Within- and among- winter repeatability (R) of distance from the Isle of May at which adult colour-ringed shags known to have bred on the Isle of May, and with an interval between first and last resightings that falls within the population-wide interquartile range, were resighted during winters 2009-2012.

**Winter**	**R_{all}**	**R_{res}**	**V_i**	**V_t**	**p**	**No. individuals**	**No. sightings**	**No. sites**	**Distance range (km)**
2009-2010	0.79	0.88	14191	16080	0.001	75	263	12	427
2010-2011	0.81	0.80	9905	12446	0.001	178	593	17	676
2011-2012	0.89	0.86	13567	15749	0.001	262	729	19	512
Among winters	0.68	0.68	8948	14101	0.001	93	279	16	408

Repeatabilities were estimated across the whole winter period (1st September- 31st March) for each year for individuals with an interval between the first and last resighting within the interquartile range of intervals for the whole population (R_{res}). V_i and V_t are the within-individual and total variances of resighting distance from the Isle of May respectively, and p values are the probability of the estimated R_{chick} values occuring by chance. The numbers of individuals, resightings and sites are the totals included in each analysis, with “sites” defined for descriptive purposes as the number of known roosts separated by ≥1km. Distance range is the maximum coastline distance covered. Repeatabilities calculated across all resighted individuals are also shown for comparative purposes (R_{all}, see also Table 6).
