# Supplementary material for: Site Fidelity and Individual Variation in Winter Location in Partially Migratory European Shags
Source: PLoS One. 2014 Jun 3;9(6):e98562. doi: 10.1371/journal.pone.0098562 (PMC4043777; doi:10.1371/journal.pone.0098562)
Supplement: Table S3 — Numbers of individual colour-ringed adult shags that were known to have bred on the Isle of May that were resighted at each winter survey site during winters 2009–2010, 2010–2011 and 2011–2012. (PDF) [file pone.0098562.s010.pdf]

Table S3. Numbers of individual colour-ringed adult shags that were known to have bred on the Isle of May that were resighted at each winter survey site during winters 2009-2010, 2010-2011 and 2011-2012.

		**2009-2010**		**2010-2011**		**2011-2012**	
**Site number**	**Distance from the IOM (km)**	**No. individuals**	**No. resightings**	**No. individuals**	**No. resightings**	**No. individuals**	**No. resightings**
**1**	486	2	2	0	0	0	0
**2**	376	2	2	9	18	7	24
**3**	328	0	0	1	1	0	0
**4**	298	0	0	0	0	5	5
**5**	291	43	154	49	156	55	216
**6**	245	6	8	3	3	15	23
**7**	239	5	5	3	3	8	8
**8**	234	1	1	3	3	1	1
**9**	231	5	11	17	22	21	30
**10**	228	0	0	2	2	0	0
**11**	226	19	61	30	201	45	273
**12**	221	0	0	5	8	9	17
**13**	204	0	0	16	31	4	4
**14**	198	13	20	11	16	0	0
**15**	196	8	10	1	1	33	95
**16**	191	6	12	3	3	8	8
**17**	147	1	10	2	4	4	19
**18**	63	3	3	0	0	0	0
**19**	14	3	3	19	20	1	1
**20**	8	0	0	38	40	108	127
**21**	7	151	257	179	300	192	317
**22**	**0**	**118**	**130**	**386**	**624**	**268**	**398**
**23**	-19	1	1	3	4	3	5
**24**	-32	1	1	1	1	7	22
**25**	-34	0	0	2	3	5	12
**26**	-58	0	0	1	1	0	0
**27**	-88	0	0	6	7	0	0
**28**	-136	2	9	2	16	3	4

Sites defined for descriptive purposes as the number of roosts separated by ≥ 1km (Figure 1). Positive and negative values are distances north and south of the Isle of May (highlighted in bold) respectively.
